# Supplementary material for: Comparative Characterization of Plasmodium falciparum Hsp70-1 Relative to E. coli DnaK Reveals the Functional Specificity of the Parasite Chaperone
Source: Biomolecules. 2020 Jun 4;10(6):856. doi: 10.3390/biom10060856 (PMC7356358; doi:10.3390/biom10060856)
Supplement: Supplementary file 1 [file biomolecules-10-00856-s001.pdf]

Supplementary Materials:

Table S1. ATPase kinetics of DnaK, PfHsp70-1 and KPf

Proteins	V_{max} (n/mol/min/mg)	K_m (μM)
DnaK	14.33 (± 1.4) *	179.1 (± 3.7)
DnaK+ PfHsp40	19.65 (± 0.81)	354.6 (± 8.1)
PfHsp70-1	22.45 (± 1.8)	370.8 (± 3.8)
PfHsp70-1+ PfHsp40	34.50 (± 0.62)*	34.17 (± 0.5)
KPf	27.6 (± 0.22)	29.58 (± 2.2)
KPf + PfHsp40	42.6 (± 2.1)**	25.65 (± 0.23)

Table legends: The respective *Vmax* and *Km* values are shown. Standard deviations shown represent three independent assays made using separate protein purification batches. Statistical significance of data was validated by one way ANOVA test ($p < 0.05$ *) and ($p < 0.01$**).

Table S2. Comparative ATP binding affinities for the Hsp70 proteins

Protein	ATP K_D /μM (± standard deviation)
KPf	0.38 (± 0.9)*
PfHsp70-1	3.52 (± 1.1)
DnaK	83.80 (± 6.6)

The table shows equilibrium constant (K_D) values representing the ATP binding affinities for DnaK, KPf, and PfHsp70-1. The ligand was the immobilized protein mounted onto HTE chip surface, while ATP represented the analyte which was injected at a flow rate of 100 μl/min. The standard deviations shown in parenthesis were obtained from three independent assays and validated using one way ANOVA test (p<0.05*).

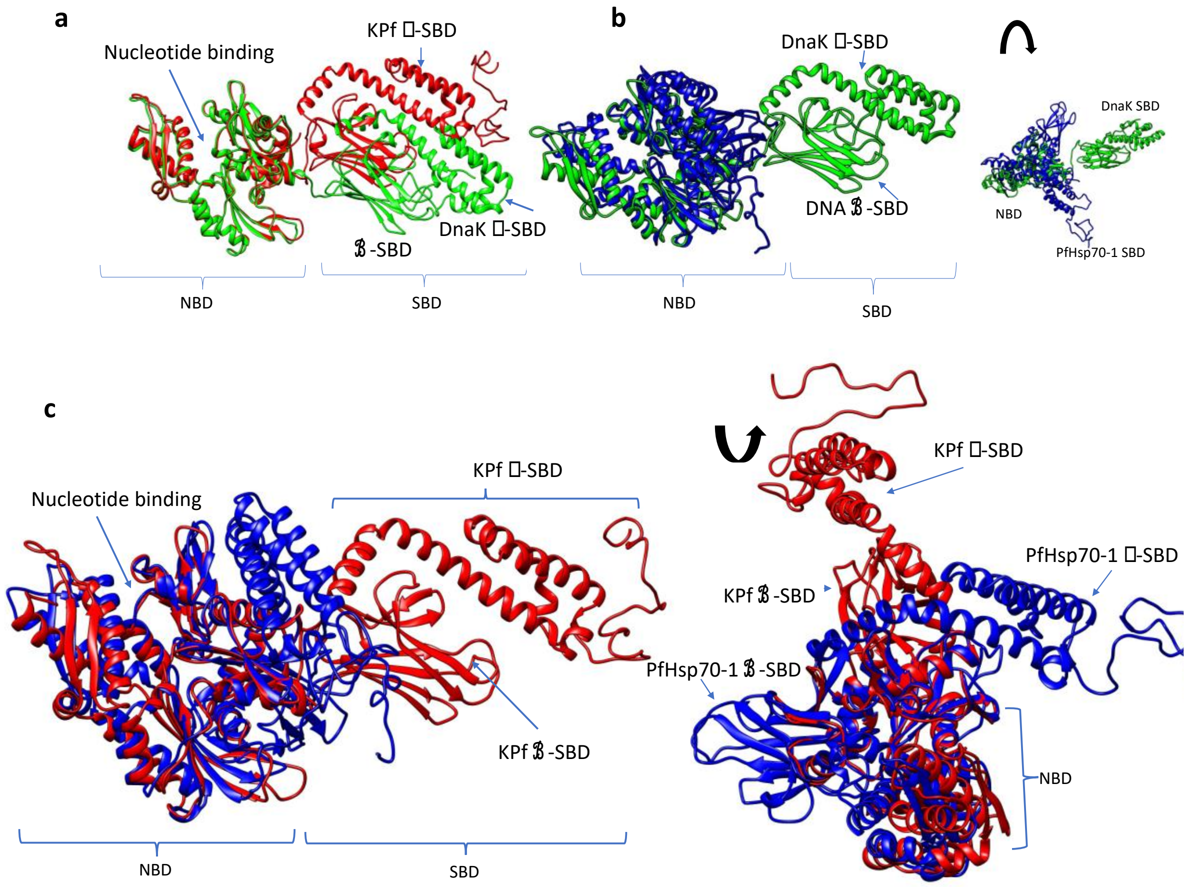

Figure S1. Homology models of the Hsp70s.

The three-dimensional models for Hsp70s were compared by superimposing them. The comparison structure of the nucleotide binding domain (NBD) and the substrate binding domain (SBD) for (**a**) DnaK (green) versus KPf (red); (**b**) DnaK (green) versus PfHsp70-1(Blue) and also shown is an insert representing top view showing domain orientation (**c**) PfHsp70-1 (blue) versus KPf (red) and also shown is an insert representing top view of the model. The SBD is subdivided into the peptide binding (β-SBD) and the lid (α-SBD). Models were generated using (2KHO) [33] as template.

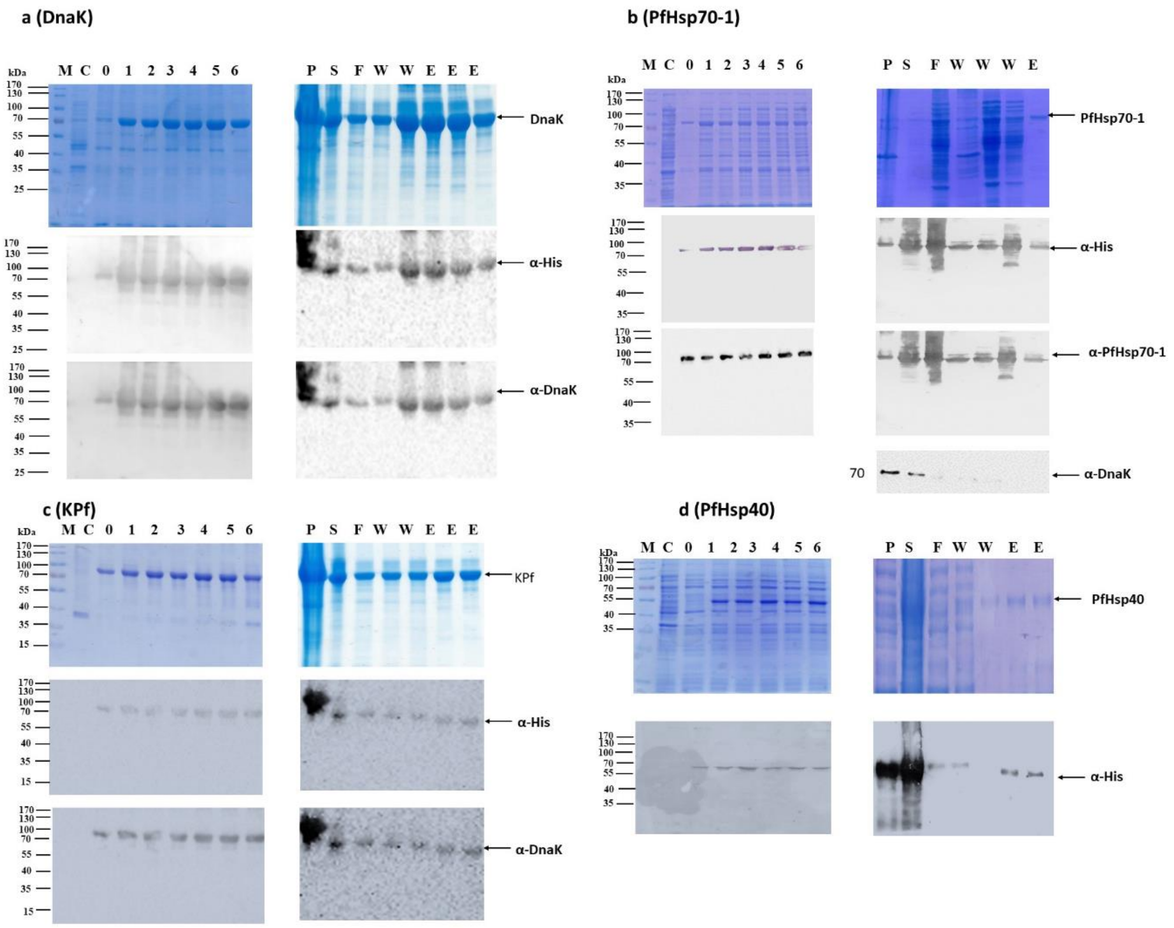

Figure S2. Expression and purification of recombinant Hsp70s. The respective recombinant proteins were expressed in *E. coli* XL1 Blue cells, purified using affinity chromatography and were analysed with SDS-PAGE (12 %): (**a**) DnaK; (**b**) PfHsp70-1; (**c**) KPf and (**d**) PfHsp40. Various lanes are denoted as follows: M–Page ruler (Thermo Scientific) in kDa; C—total extract for cells transformed with a neat pQE30 plasmid as control; 0–total extract of cells transformed with pQE30/PfHsp70-1/KPf/DnaK/PfHsp40 prior to IPTG induction; 1 - 6 - total cell lysate obtained hourly up to 6 h post induction; P, S; pellet and soluble fractions obtained from the total lysate of cells transformed with pQE30/PfHsp70-1/KPf/DnaK/PfHsp40, respectively; F- flow though, W-wash fraction; E-eluted fraction.

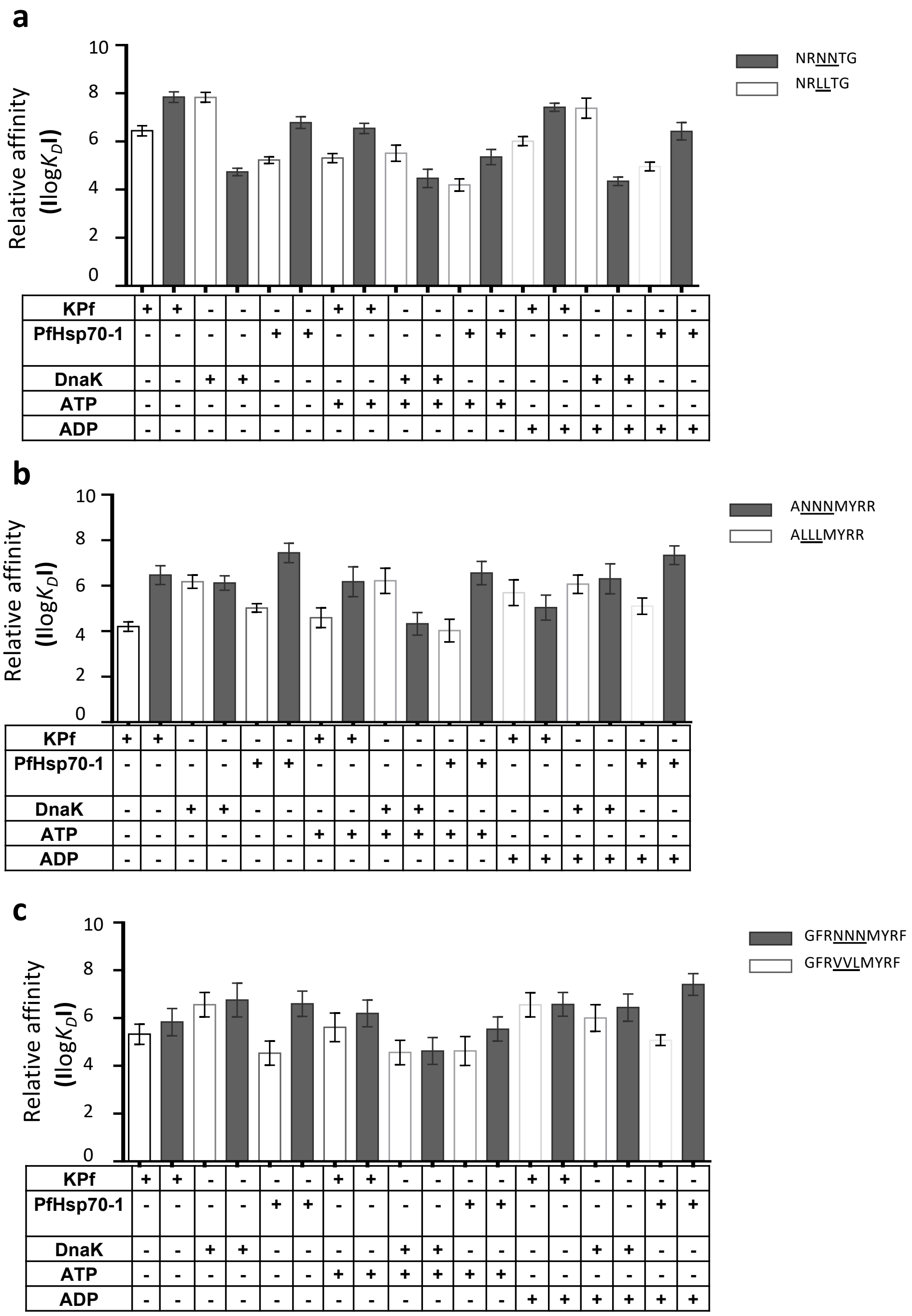

Figure S3. The relative affinities of DnaK, KPf and PfHsp70-1 for the various peptides were determined in the presence or absence of 5 mM ATP/ADP. Comparative relative affinities for peptides: (**a**) NRLLTG and NRNNTG (**b)** ALLLMYRR and ANNNMYRR; (**c)** GFRVVLMYRF and GFRNNNMYRF are depicted as bar graphs. The error bars shown were generated from three assays conducted using independent Hsp70 protein preparations.

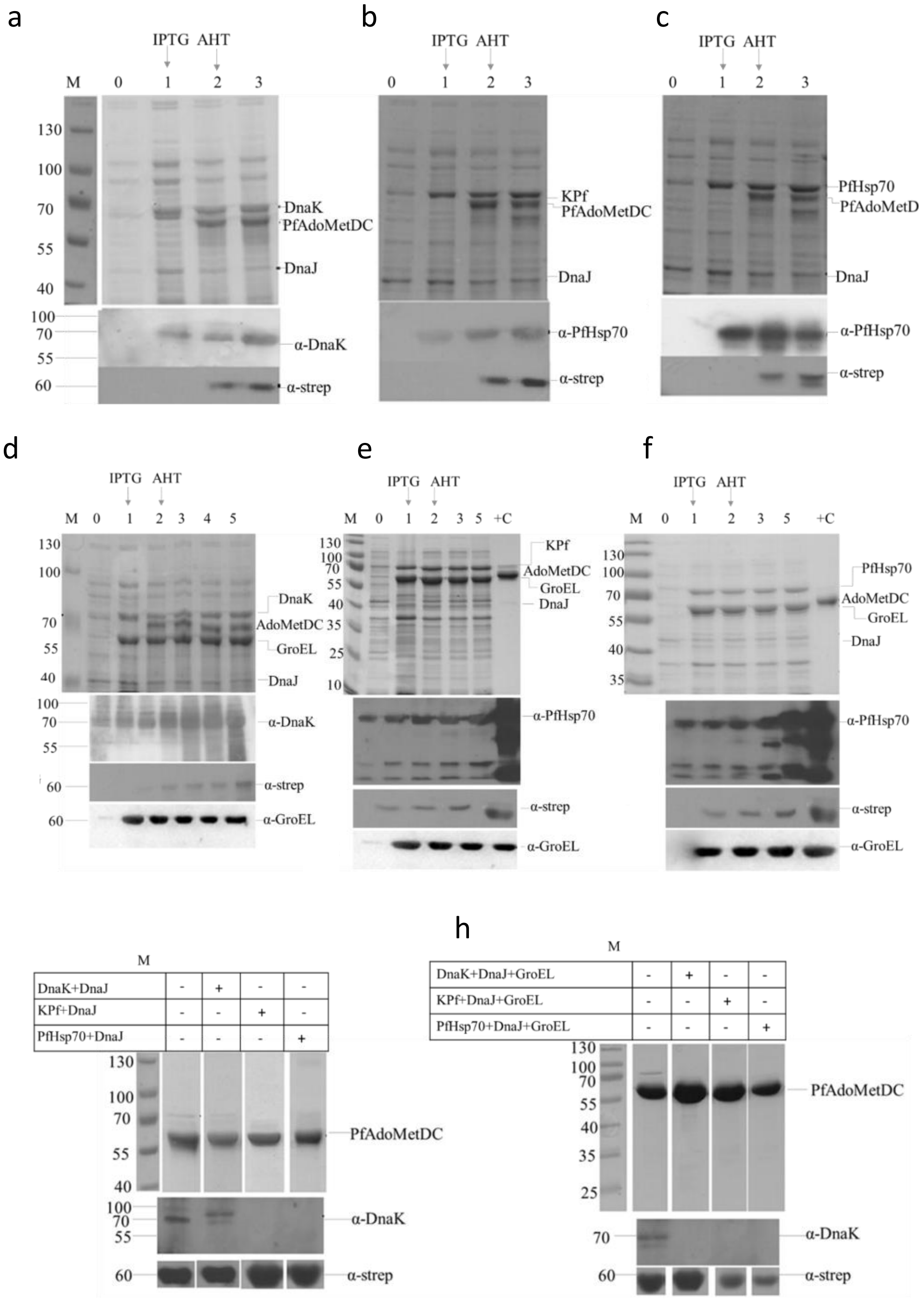

Figure S4: Co-expression and purification of PfAdoMetDC. The PfAdoMetDC recombinant proteins were co-expressed with different chaperone sets in *E. coli* BL21 Star (DE3) cells and were analysed by SDS-PAGE (12 %) and Western blotting. Various co-expression chaperone sets were as follows: (a) DnaK+DnaJ; (b) KPf+DnaJ; (c) PfHsp70+DnaJ; (d) DnaK+DnaJ+GroEL; (e) KPf+DnaJ+GroEL; (f) PfHsp70+DnaJ+GroEL. Purification of PfAdoMetDC purified following expression with the various chaperone combinations is shown: (g); (h). Various lanes are denoted as follows: M–Page ruler (Thermo Scientific) in kDa; 0 – 5 total cell lysate obtained 0-5 hours IPTG induction; Note, induction with AHT was done 1 hour after IPTG induction. ''+C'' represents the positive control (purified PfAdoMetDC).

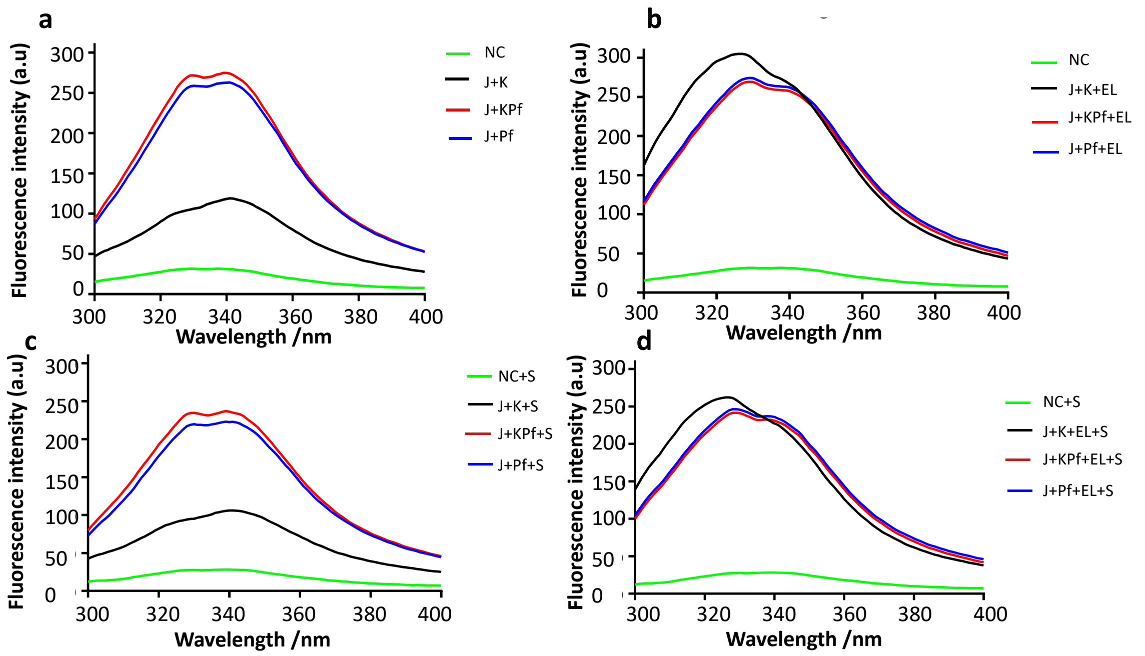

Figure S5. Combined tryptophan and tyrosine florescence spectra of PfAdoMetDC

Shown are the respective intrinsic combined tryptophan and tyrosine fluorescence signals of PfAdoMetDC co-expressed with: (a) Hsp70-DnaJ; (b) Hsp70-DnaJ-GroEL chaperone, respectively. PfAdoMetDC was analysed either in the absence of the substrate, SAM (c) and in the presence of SAM (d), respectively. The chaperone sets are denoted as: PfHsp70+DnaJ (J+Pf); PfHsp70+DnaJ+GroEL (J+Pf+EL); KPf+DnaJ (J+KPf); KPf+DnaJ+GroEL (J+KPf+EL). PfAdoMetDC produced in the absence of supplementary chaperones is represented by 'NC'.
